# Geographical origin impact on volatile composition and some quality parameters of virgin olive oils extracted from the “Ayvalık” variety

**DOI:** 10.1016/j.heliyon.2020.e04919

**Published:** 2020-09-16

**Authors:** Didar Üçüncüoğlu, Dilek Sivri-Özay

**Affiliations:** a Cankiri Karatekin University, Faculty of Engineering, Department of Food Engineering, 18-100, Cankiri, Turkey; b Hacettepe University, Faculty of Engineering, Department of Food Engineering, 06-850, Ankara, Turkey

**Keywords:** Food science, Virgin olive oil, Food analysis, Volatile compounds, Oxidative stability, Fatty acid composition, Agriculture, Ayvalik olive cultivar, Natural product chemistry

## Abstract

"Ayvalık" is one of the prominent olive cultivar used for producing virgin olive oil (VOO) in Turkey. In this study, 215 olive samples of "Ayvalık" were harvested from 14 different locations in North Aegean Region of Anatolia by hand-picking during three consecutive crop seasons. The early harvested cold press VOO samples were produced at lab-scale and the quality indices (free acidity, peroxide value and spectral absorption at 232, 266, 270 and 274 nm), induction time, colour values, fatty acid and volatile profiles were determined in order to examine changes on composition of the "Ayvalık" olive oils based on their growing area. Characteristically, it was found that volatile fraction of "Ayvalık" VOOs composed of aldehydes (29.72), terpene (12.68), alcohol (11.65), benzene ringed compound (4.71), ketone (3.49), organic acid (2.87), ester (1.84), furan (0.96) compounds on average percentage. It was highlighted with this research 61.84–87.36% of aldehydes, 0.00–91.11% of ketones, 0.00–46.11% of esters, and 34.53–92.06% of alcohols were generated only by lipoxygenase pathway. As a conclusion, Ayvalık VOOs had different chemical composition based on geographic origin. Therefore, it was considered that this work is so promising to directly accelerate that the number of geographic indicated VOOs linked to "Ayvalık" cultivar.

## 1 Introduction

The consumption of virgin olive oil (VOO) all over the world has reached a rising trend due to its high nutritional value and functional compounds such as monounsaturated fatty acid, tocopherols, phenolics and sterols. The presence of these bioactive constituents in VOO is directly influence on its oxidative stability, shelf life and sensory properties (Hernáez et al., 2019; Sanaeifar and Jafari, 2019). Numerous epidemiological and clinical studies approved that Mediterranean diet covering regular consumption of VOO reduce the incidence of metabolic disorders such as heart disease, stroke and Type-II diabetes (Corominas-Faja et al., 2018; Soto-Alarcon et al., 2018; Tsartsou et al., 2019).

The olive oils, extracted from *Olea europaea* L. fruit, can be classified as extra virgin olive oil (EVOO), virgin olive oil (VOO) and lampant olive oil (LOO) depending on its chemical (i.e. free acidity, peroxide value, UV absorptions) and sensorial (i.e. odor, taste, color, appearance) quality according to the European Union regulations (EEC, 2016).

Turkey is the third olive producer of the world (FAOSTAT, 2020). "Ayvalık" (Edremit Yağlık) is the eminent olive cultivar (cv.) which utilized for oil production in Turkey. Ayvalık cv. is a quarter of the olive trees in Aegean Region (TURKPATENT, 2020). However, this cultivar is more widespread in North Aegean Region; it was started to grown in other parts of Anatolia such as Antalya, Mersin, and Adana recently (Naskali, 2016). Therefore, several researchers focused on "Ayvalık" oils quality and purity parameters. Classifying geographic origins of "Ayvalık" VOOs topic was examined before several researchers based on fatty acid profile (Dıraman et al., 2011; Oğraş et al., 2016; Ucuncuoglu, 2019) and phenolic profiles (Alkan et al., 2012). To the best of current knowledge, there was no research about geographical discrimination based on volatile profile and quality parameters of "Ayvalık" VOOs in Turkey.

Sensorial profile (aroma) is a crucial quality parameter for EVOO and depends on volatile and semi-volatile fractions. The volatile fraction of VOO is principally generated from the degradation pathways of linoleic and linolenic acids by various enzymes, mainly lipoxygenase. These reactions release when malaxation starts after crushing. Generally, positive notes and desired volatiles occur by this pathway. The other volatiles can be formed by direct fatty acid metabolism, as well as by sugar fermentations or amino acid conversion. The former reactions produce linear acids (i.e. acetic acid), alcohols (i.e. ethyl alcohol), esters (i.e. ethyl acetate) and ketones (i.e. propanone). The latters can generate branched esters, aldehydes, alcohols and acids etc. The desirable aroma compounds are attributed to C6 and C5 volatiles, including aldehydes, esters, alcohols, hydrocarbons, furan and ketones. There are some other volatile generation pathways caused from lipid oxidation mechanisms, microbial fermentation reactions or damaged olive fruit, which gives defective or off-flavours to VOO ketones (Kalua et al., 2007; Boskou, 2012).

**Figure 1:**
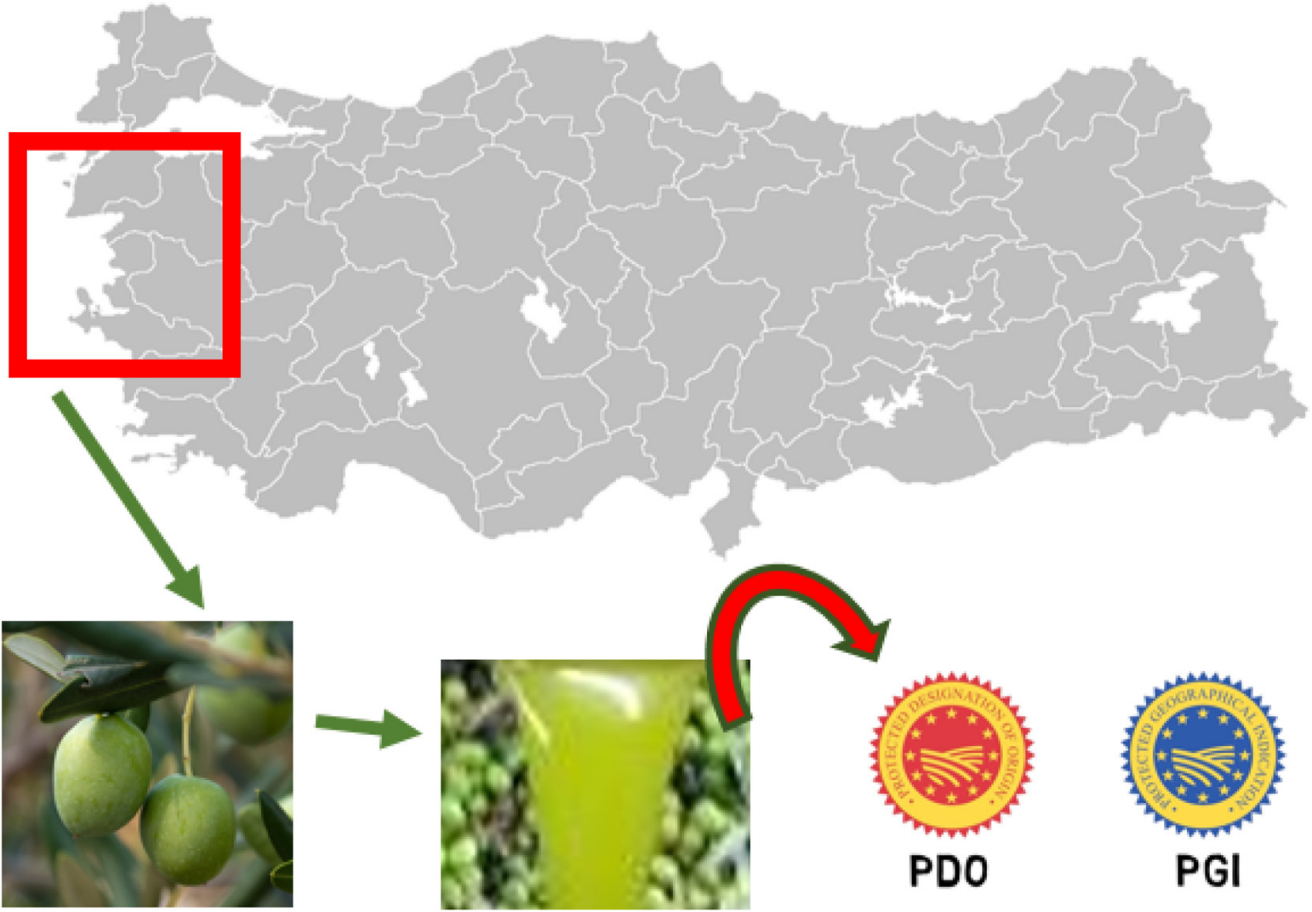
Graphical abstract.

The volatile profile of VOO and its sensorial descriptions affects the consumer preferences and so rural economy. Therefore, volatile compounds previously used for registering "Geographical Indications" such as Protected Designation of Origin (PDO) and Protected Geographical Indications (PGI) by several authorities (Barbieri et al., 2019). A geographical indication is a quality-sign correspond with a specific producing method, and its quality characteristics linked with its geographical origin or growing area. Regarding olive oils, the EU Regulation has made a labelling with geographical origin information compulsory (Likudis, 2016). The main goal of this study was to contribute to the creation of a comprehensive "Ayvalık" VOOs database via examining chemical composition of VOOs harvested from 14 different growing areas in Northern Aegean region during three consecutive crop seasons. The graphical abstract was shown at Figure 1.

## 2 Materials and methods

### 2.1 Materials and sample modelling

The olive samples belongs to "Ayvalık" cv. (n: 215) were harvested from 14 different geographic origins of North Aegean Region at early stages (4th week of October-1st week of November) of maturation during three consecutive harvest year by hand-picking. The number of sample at first harvest on-year was 76, at second harvest off-year was 37 and at third harvest on-year was 102.

**Figure 2:**
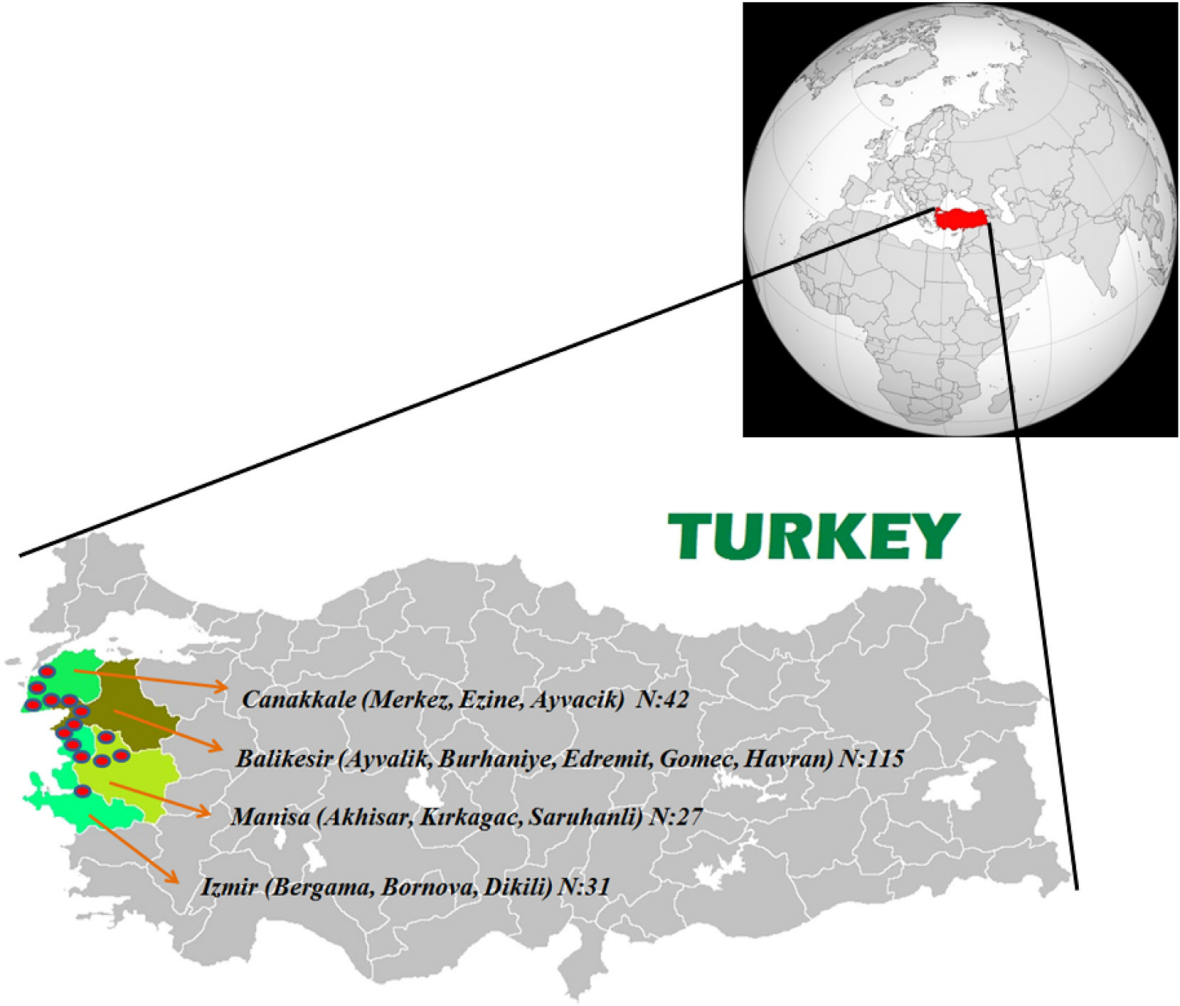
Geographic origins of monocultivar (Ayvalık cv.) olives. GPS Coordinates: 38°1′17.73″-40°10′9.06″ North Latitudes and 26°10′49.96″-27°58′16.10″ East Longitudes.

Edremit (GO1), Gömeç (GO2), Ayvalık (GO3), Havran (GO4) and Burhaniye (GO5) were the sub-locations from Balıkesir; Ayvacık (GO6), Merkez (GO7) and Ezine (GO8) were the sub-locations from Çanakkale; Bergama (GO9), Bornova (GO10) and Dikili (GO11) were the sub-locations from İzmir, and finally, Akhisar (GO12), Kırkağaç (GO13) and Saruhanlı (GO14) were the sub-locations from Manisa cities. Geographic origins of monocultivar olives and number of samples were detailed in Table 1 and shown at Figure 2. The mathematical coordinates of the investigated geographic area was between 26-27° East Longitudes and 38–40° North Latitudes.

**Table 1:** Geographic origins and sample numbers of monocultivar olives (Ayvalık *cv.)*.

Code	Geographic Origins	First Harvest Year (On-Year)	Second Harvest Year (Off-Year)	Third Harvest Year (On-Year)	Total
GO1	Balıkesir-Edremit	9	19	15	**43**
GO2	Balıkesir-Gömeç	6	3	9	**18**
GO3	Balıkesir-Ayvalık	9	2	9	**20**
GO4	Balıkesir-Havran	8	1	5	**14**
GO5	Balıkesir-Burhaniye	9	3	8	**20**
GO6	Çanakkale-Ayvacık	9	-	11	**20**
GO7	Çanakkale-Merkez	-	2	-	**2**
GO8	Çanakkale-Ezine	9	2	9	**20**
GO9	Izmir-Bergama	-	-	6	**6**
GO10	Izmir-Bornova	-	-	3	**3**
GO11	Izmir-Dikili	6	3	13	**22**
GO12	Manisa-Akhisar	11	2	5	**18**
GO13	Manisa-Kırkağaç	-	-	3	**3**
GO14	Manisa-Saruhanlı	-	-	6	**6**
**Total**		**76**	**37**	**102**	**215**

Edremit (GO1), Gömeç (GO2), Ayvalık (GO3), Havran (GO4) and Burhaniye (GO5) were the sub-locations from Balıkesir; Ayvacık (GO6), Merkez (GO7) and Ezine (GO8) were the sub-locations from Çanakkale; Bergama (GO9), Bornova (GO10) and Dikili (GO11) were the sub-locations from İzmir, and finally, Akhisar (GO12), Kırkağaç (GO13) and Saruhanlı (GO14) were the sub-locations from Manisa cities.

Approximately 5 kg olives were harvested and were immediately transported to the laboratory in the mesh-bags. The leaves were removed and the healthy olives were washed to obtain oil samples using a laboratory scale physical extraction unit (HAUS Centrifuge Technologies, Aydin, Turkey) including a crusher, a malaxer and a centrifuge without any delay. Malaxation process was performed for 30 min at 25 °C. A vertical centrifuge (3000 rpm, 2 min) was used for solid-liquid phase separation. Samples were passed through cotton filter and stored at + 4 °C till analysis.

Every VOO sample was divided into three amber bottles (50 ml) without any headspace on each. The first bottle was used for only volatile and fatty acid profile analysis; the second was used for other chemical analyses. The last bottle was kept in the fridge to be used if necessary.

### 2.2 Methods for determining quality parameters

Free acidity (as % oleic acid), peroxide value (as meq active O_2_/kg oil), K_232_ and K_270_ values were determined according to IOC (2015a, 2015b; 2016). Fatty acid profile was determined according to IOC method. Fatty acids methyl esters (FAMEs) were prepared according to COI/T.20/Doc. No. 24 (IOC, 2001) and they were analysed by gas chromatography (Thermo Scientific, Trace™ Ultra Gas Chromatograph, Waltham, USA) equipped with a 100 m long capillary column (0.25 mm id, 0.20 μm film thickness) and a flame ionization detector (FID).

### 2.3 Methods for determining colour indices and oxidative stability

*L*, *a*∗ and *b*∗ values were measured with Minolta Spectrophotometer (CM-3600d, Japan) as lightness, red-green and blue-yellow colour. Induction period was measured with the Rancimat 743 (Metrohm, Basel, Switzerland) apparatus at 120 °C with a continuous airflow of 20 L.h^−1^ passing through the oil samples. Induction time (as hour) is defined at the point of rapid change in the rate of oxidation resulting in a sharp inflection point on the oxidation curve.

### 2.4 Method for determining volatile profile

Volatile compounds were determined using 50/30μm thickness DVB/CAR/PDMS three-phase fiber (2 cm) with DHS-SPME (Supelco Co., Bellefonte, CA, USA), GC-MS (Thermo Scientific GC, DSQ II Series Single Quadruple GC/MS, Waltham, MA, USA). TR Wax MS 60 capillary column (60 m × 0.32 mm i.d. × 0.5 μm thickness) was coupled to GC. *4-methyl-2-pentanol* (Aldrich, Germany) was used as an internal standard. VOO (3 g) was added to a 15 ml vial with 1.5 ppm internal standard. The vial was then closed with a polytetrafluoro ethylene (PTFE) septum. The fibre was exposed for 30 min at 40 °C, after 30 min at 40 °C for equilibration time. Thermal desorption time was 5 min for injection. The temperature was set at 40 °C for 10 min, followed by an increase of 3 °*C min*-1 up to 240 °C, and then held for 15 min. The injector was kept at 240 °C. Helium was used as carrier gas (1.0 mL.min^−1^). The scanning mass range varied from 50 to 550 m/z. Mass spectra were recorded at 70 eV. All measurements were triplicated. Before use, the fiber was conditioned at 260 °C for 1 h. Moreover, a blank test (both empty vial and fiber) was carried out before every run-day to prevent the release of undesirable compounds. The identification of volatile compounds was performed by comparing their retention time with NIST and Wiley library tools, and by checking mass isotope distributions.

### 2.5 Data processing

Statistical analysis was performed by SPSS (version 23, IBM SPSS Statistics Inc. Chicago, IL) statistical software using One-way ANOVA method. Differences among all groups were determined by Duncan test at 95% confidence level.

## 3 Results and discussion

### 3.1 Quality parameters, colour indices & oxidative stability

It was determined that free acidity values were altered between 0.3% and 0.6%; peroxide values 3.0 and 10.1; K_232_ values 1.6 and 2.1; and, K_270_ values 0.1 and 0.2 among the samples (Table 2). Quality parameters data obviously suggested that all investigated samples in this study were "extra virgin" category according to IOC (2015a). GO2 and GO11 were significantly different in terms of free acidity; GO4 and GO14 in terms of peroxide value; GO11 in terms of K_232_; GO8, and GO11 & GO14 in terms of K_270_ (P ≤ 0.05).

**Table 2:** Quality parameters, color indices & oxidative stability (induction period) of Ayvalık VOOs.

Locations	Free Acidity	Peroxide Value	K232	K270	L∗	a∗	b∗	Induction Period
GO1	0.5 ± 0.02 abc	9.2 ± 0.60 ab	2.0 ± 0.06 a	0.2 ± 0.01 abc	32.9 ± 0.41 b	1.2 ± 0.07 a	8.2 ± 0.40 abc	7.2 ± 0.34 ab
GO2	0.6 ± 0.04 a	8.7 ± 0.60 abc	2.0 ± 0.10 a	0.2 ± 0.01 abc	32.5 ± 0.77 b	1.2 ± 0.17 a	7.5 ± 0.91 bc	6.1 ± 0.39 b
GO3	0.4 ± 0.04 bcde	7.8 ± 0.53 abc	2.1 ± 0.05 a	0.1 ± 0.01 bcd	33.2 ± 0.58 b	0.8 ± 0.10 ab	9.8 ± 0.80 ab	6.8 ± 0.68 ab
GO4	0.3 ± 0.03 de	10.1 ± 0.75 a	2.0 ± 0.06 a	0.2 ± 0.01 abc	32.8 ± 0.42 b	0.8 ± 0.14 ab	7.8 ± 0.75 abc	7.0 ± 0.44 ab
GO5	0.4 ± 0.04 abcde	8.7 ± 0.58 abc	2.1 ± 0.10 a	0.2 ± 0.01 ab	33.6 ± 0.51 b	1.0 ± 0.15 ab	8.8 ± 0.58 abc	8.3 ± 0.60 ab
GO6	0.4 ± 0.03 cde	8.4 ± 0.77 abc	1.8 ± 0.07 ab	0.1 ± 0.01 bcd	31.6 ± 0.60 b	0.7 ± 0.10 ab	6.9 ± 0.68 bc	8.4 ± 0.99 ab
GO7	0.4 ± 0.03 abcd	8.9 ± 0.86 ab	2.1 ± 0.19 a	0.1 ± 0.01 bcd	32.1 ± 0.54 b	1.0 ± 0.08 ab	8.6 ± 0.56 abc	5.8 ± 0.56 b
GO8	0.6 ± 0.04 ab	9.1 ± 1.29 ab	2.0 ± 0.00 a	0.2 ± 0.00 a	36.1 ± 0.10 a	0.6 ± 0.04 b	10.9 ± 0.54 a	5.7 ± 0.68 b
GO9	0.3 ± 0.02 de	9.0 ± 0.50 ab	1.9 ± 0.08 a	0.1 ± 0.01 cd	32.4 ± 0.62 b	1.1 ± 0.11 ab	8.6 ± 0.66 abc	7.2 ± 0.62 ab
GO10	0.5 ± 0.05 abc	6.0 ± 0.72 bcd	2.0 ± 0.08 a	0.1 ± 0.01 bcd	31.3 ± 0.58 b	0.9 ± 0.13 ab	8.3 ± 1.10 abc	7.3 ± 0.39 ab
GO11	0.3 ± 0.07 e	6.0 ± 0.72 bcd	1.6 ± 0.02 b	0.1 ± 0.01 d	28.8 ± 0.82 c	0.6 ± 0.08 b	3.9 ± 1.39 d	7.4 ± 1.30 ab
GO12	0.4 ± 0.03 abcde	8.0 ± 0.67 abc	2.0 ± 0.09 a	0.2 ± 0.01 abc	33.3 ± 0.46 b	0.8 ± 0.12 ab	8.9 ± 0.56 abc	8.1 ± 0.29 ab
GO13	0.5 ± 0.04 abc	5.5 ± 0.75 cd	2.1 ± 0.07 a	0.1 ± 0.01 cd	31.4 ± 0.74 b	1.0 ± 0.16 ab	6.5 ± 1.13 cd	8.4 ± 1.79 ab
GO14	0.3 ± 0.04 de	3.0 ± 0.73 d	1.8 ± 0.05 ab	0.1 ± 0.01 d	32.2 ± 0.26 b	1.1 ± 0.15 ab	11.0 ± 0.54 a	9.5 ± 1.12 a

The values were expressed as mean ± standard deviations. Means within a column with different letters are significantly different (P ≤ 0.05). Free acidity was given as % g oleic acid; peroxide value as meq active O2/kg oil; induction period as hour, respectively.

Induction period, is an indicator of oxidative stability, changed in a wide range from 5.7 to 9.5 h and GO2, GO7, GO8 and GO14 had different oxidative stability (P ≤ 0.05). The lowest induction period was measured at GO8 and the highest was at GO14. GO7 and GO8 were classified with lower induction period from the other geographic origins (Table 2).

*L*∗ values (lightness) were changed from 28.8 to 36.1, *a*∗ values (redness) 0.6 to 1.2, and *b*∗ values (yellowness) 3.9 to 11.0 indicating that colours altered from green to light yellow. GO8 and GO11 were differed in terms of *L*∗ values, GO1 & GO2 and GO8 & GO11 in terms of *a*∗ values, GO8 & GO14, and GO11 in terms of *b*∗ values (Table 2). Even though colour is not regarded as an important quality characteristic for VOOs, it has a great effect on consumer acceptance. Our results were in agreement with both regulations for EVOO and reported studies (Kıralan and Bayrak, 2013; Toker et al., 2016; Guclu et al., 2016) before related to "Ayvalık" EVOOs except *b*∗ values.

In this study, it was found that the major fatty acids were oleic (61–70%), palmitic (12–16%), linoleic (10–16%), stearic (2–4%) and palmitoleic (1–3%) acids, respectively. Previously reported researches (Andjelkovic et al., 2009; Gurdeniz et al., 2010; Dıraman et al., 2011) showed that Ayvalık VOOs had 60–77% oleic, 10–19% palmitic, 8–17% linoleic, 2–4% stearic and 0.5–2% palmitoleic acids, respectively. According to C18:1 content, it was observed that two main cluster, one of them includes GO3, GO9, GO10, GO11, GO13 and GO14 origins which had 67–69% oleic acid. The other group was heavily contained 61–64% oleic acid. The most distinct origins were GO5 and GO14 by palmitic acid. GO10 had the lowest and GO8 had the highest linoleic acid percentage. GO1, GO2, GO9 and GO10 origins were significantly distinguished among others in terms of palmitoleic acid content (P ≤ 0.05). Fatty acid compositions of "Ayvalık" VOOs were presented in Table 3. It was shown a good (P ≤ 0.05) statistic description concerning each geographical origin (Table 3). For example, among Balıkesir sub-locations, GO1 and GO2 were characterized by the lowest C16:1 and the highest C18:0 contents. Moreover, GO3 was separated from the others based on the lowest saturated fatty acids such as C16.0 (12.5%) and C18:0 (2.3%), and the highest C18:1 (67.5%) and C18:3 (0.9%) contents. GO6 origin was the only different sub-location among Çanakkale based on both arachidic and eicosanoic fatty acids. Among Izmir sub-locations, GO10 was distinguished by myristic and eicosanoic acids, and GO11 by palmitoleic and margaric acids statistically. GO12 was also statistically varied among Manisa sub-locations in terms of C16:0, C17:1, C18:1 and C18:2.

**Table 3:** Fatty acid composition of Ayvalık VOOs.

Locations	C14:0	C16:0	C16:1	C17:0	C17:1	C18:0	C18:1	C18:2	C18:3	C20:0	C20:1	C24:0
GO1	0.0 ± 0.00bcd	15.2 ± 0.28ab	1.4 ± 0.21b	0.1 ± 0.01ab	0.2 ± 0.01ab	3.7 ± 0.21ab	62.8 ± 0.52c	15.0 ± 0.25ab	0.6 ± 0.03bc	0.4 ± 0.01bcd	0.3 ± 0.01bc	0.1 ± 0.01ab
GO2	0.0 ± 0.01a	15.1 ± 0.14ab	1.2 ± 0.20b	0.2 ± 0.01a	0.2 ± 0.00a	4.2 ± 0.07a	62.9 ± 0.54c	14.7 ± 0.29abc	0.5 ± 0.03bc	0.4 ± 0.00bcd	0.3 ± 0.01ab	0.1 ± 0.00abc
GO3	0.0 ± 0.00d	12.5 ± 0.02d	2.1 ± 0.00a	0.1 ± 0.00c	0.1 ± 0.00f	2.3 ± 0.01e	67.5 ± 0.06a	13.6 ± 0.03bcde	0.9 ± 0.02a	0.4 ± 0.01d	0.3 ± 0.00ab	0.1 ± 0.00d
GO4	0.0 ± 0.00bcd	14.7 ± 0.31bc	2.1 ± 0.08a	0.1 ± 0.00bc	0.1 ± 0.00def	2.7 ± 0.09de	64.3 ± 0.46bc	14.4 ± 0.26abcd	0.6 ± 0.02bc	0.4 ± 0.01bcd	0.3 ± 0.00ab	0.1 ± 0.00cd
GO5	0.0 ± 0.00bc	16.2 ± 0.38a	2.4 ± 0.11a	0.2 ± 0.01ab	0.2 ± 0.01bcd	2.3 ± 0.03e	62.3 ± 0.28c	14.8 ± 0.33abc	0.6 ± 0.04bc	0.4 ± 0.02bcd	0.3 ± 0.01ab	0.1 ± 0.00abcd
GO6	0.0 ± 0.01b	14.9 ± 0.26ab	2.2 ± 0.17a	0.1 ± 0.01bc	0.1 ± 0.01def	3.6 ± 0.18ab	63.0 ± 0.59c	14.7 ± 0.45abc	0.5 ± 0.03bc	0.4 ± 0.02cd	0.2 ± 0.01d	0.1 ± 0.01bcd
GO7	0.0 ± 0.00bcd	15.9 ± 0.27ab	2.1 ± 0.24a	0.1 ± 0.01c	0.1 ± 0.01cde	3.4 ± 0.13bc	61.2 ± 0.44c	15.4 ± 0.52ab	0.7 ± 0.04b	0.4 ± 0.02ab	0.3 ± 0.02ab	0.1 ± 0.01bcd
GO8	0.0 ± 0.00cd	15.4 ± 0.13ab	2.2 ± 0.15a	0.1 ± 0.00bc	0.2 ± 0.01abcd	3.5 ± 0.15ab	61.2 ± 0.44c	15.9 ± 0.34a	0.5 ± 0.03bc	0.5 ± 0.01ab	0.3 ± 0.01ab	0.1 ± 0.00abcd
GO9	0.0 ± 0.00bc	14.5 ± 0.14bc	1.4 ± 0.14b	0.1 ± 0.00bc	0.1 ± 0.00cde	2.5 ± 0.20e	67.0 ± 0.94ab	12.7 ± 0.50defg	0.6 ± 0.02bc	0.4 ± 0.02abc	0.2 ± 0.01d	0.1 ± 0.01abcd
GO10	0.0 ± 0.00d	13.4 ± 0.01cd	1.1 ± 0.01b	0.1 ± 0.00bc	0.2 ± 0.00bcd	3.3 ± 0.02bcd	69.4 ± 0.36a	10.8 ± 0.34g	0.6 ± 0.01b	0.5 ± 0.00a	0.3 ± 0.00a	0.1 ± 0.00abc
GO11	0.0 ± 0.01bc	12.6 ± 0.25d	2.7 ± 0.06a	0.2 ± 0.00a	0.2 ± 0.04abcd	2.7 ± 0.26de	67.1 ± 1.02ab	13.0 ± 0.80cdef	0.6 ± 0.08b	0.5 ± 0.04a	0.2 ± 0.01cd	0.1 ± 0.01abcd
GO12	0.0 ± 0.00bcd	14.5 ± 0.43bc	1.4 ± 0.21a	0.1 ± 0.00bc	0.2 ± 0.01abc	3.4 ± 0.12bcd	63.9 ± 1.04c	14.3 ± 0.64abcd	0.6 ± 0.05bc	0.4 ± 0.01bcd	0.3 ± 0.01ab	0.1 ± 0.01abcd
GO13	0.0 ± 0.00bc	12.8 ± 0.02d	1.4 ± 0.21a	0.1 ± 0.00c	0.1 ± 0.00ef	2.8 ± 0.02cde	68.8 ± 0.20a	11.5 ± 0.17fg	0.5 ± 0.01bc	0.4 ± 0.00cd	0.3 ± 0.00bc	0.1 ± 0.00a
GO14	0.0 ± 0.01bcd	12.2 ± 0.01d	1.4 ± 0.21a	0.1 ± 0.00bc	0.1 ± 0.01def	3.6 ± 0.06ab	67.9 ± 0.01a	12.0 ± 0.03efg	0.5 ± 0.01c	0.4 ± 0.01bcd	0.3 ± 0.00ab	0.1 ± 0.01ab

C14:0 (myristic acid), C16:0 (palmitic acid), C16:1 (palmitoleic acid), C17:0 (margaric acid), C17:1 (margoleic acid), C18:0 (stearic acid), cis-C18:1 (oleic acid), C18:2 (linoleic acid), C18:3 (linolenic acid), C20:0 (arachidic acid), C20:1 (eicosanoic acid), C22:0 (behenic acid), C24:0 (lignoceric acid). The values were expressed as mean ± standard deviations. Means within a column with different letters are significantly different (P ≤ 0.05). trans-C18:1 & C22:0 are not given at table due to statistically meaningless difference (P > 0.05).

### 3.2 The profile of volatile aroma compounds

LOX pathway includes hydroperoxide lyase, alcohol dehydrogenase, alcohol acyltransferase and isomerase enzymes which causes C5 and C6 compounds generation, in particular. Moreover, hexanal, hexan-1-ol and hexyl acetate were formed by 13-hydroperoxides of linoleic acid; cis-3-hexenal, cis-3-hexen-1-ol, cis-3-hexenyl acetate, trans-2-hexenal and trans-2-hexen-1-ol were formed by 13-hydroperoxides of linolenic acid; 2-pentanal, 2-penten-1-ol, 1-penten-3-ol, 1-penten-3-one and some other pentenyl radicals were occurred by 13-alkoxy radicals during LOX pathway. These specific compounds can be chemically grouped as aldehydes, ketones, esters and alcohols. Furthermore, these LOX products can be heavily attributed with green and fruity aroma of VOOs. Green and fruity (positive notes) odour descriptions could characterize the early stage harvesting, fresh-high quality olive fruit, and well-processing and storing conditions. The other fatty acids (both saturated and unsaturated) and nitrogen contained compounds catabolism, and biodegradation reactions linked autoxidation, thermal oxidation or microbial fermentation could be create negative notes, such as waxy, oily, winey, vinegary, musty, fermented, creamy, soapy, tallow, fried, cheesy, ethereal or mushroom-like. In this study, volatile compounds identified and quantified for "Ayvalık" VOOs. GC-MS data showed the presence of total 125 different volatile constituents in the samples. Table 4 cited that the chemical formula and generation mechanisms of volatiles, which were categorized as aldehydes, alcohols, acids, esters, ketones, terpenes, benzenoids, furans and hydrocarbons in Table 5, and their odour/flavour descriptions (Ridolfi et al., 2002; Kalua et al., 2007; Boskou, 2012; Kesen et al., 2013; Zhu et al., 2016; Song and Liu, 2018; Aparicio-Ruiz et al., 2018; Genovese et al., 2018; Abbatangelo et al., 2019; flavornet.org; pubchem.ncbi.nlm.nih.gov, The Good Scents Company Information System TGSC, 2020).

**Table 4:** Isolated volatile compounds from Ayvalık VOOs, generation pathways and sensorial descriptions.

Compound	Chemical Formula	Flavour/ Odour Description	Generation Pathway/Source
ethanol	**C**_**2**_**H**_**6**_**O**	winey, vinegary^c,d^	Anaerobic fermentation^d^
1-hexen-3-ol	**C**_**6**_**H**_**14**_**O**	woody, green plant^h^	Plant metabolite^k^
1-penten-3-ol	**C**_**5**_**H**_**10**_**O**	wet soil^b^	13-alkoxy radicals^k^
1-butanol	**C**_**4**_**H**_**10**_**O**	solvent^k^	microbial fermentation through the butanoate metabolic pathway^k^
1-heptanol	**C**_**7**_**H**_**16**_**O**	fragrant^k^	Nature of some essential oils^k^
1-pentanol	**C**_**5**_**H**_**12**_**O**	green-fruity^b^	Plant metabolite^k^
trans-2-penten-1-ol	**C**_**5**_**H**_**10**_**O**	pungent, bitter^b^	13-alkoxy radicals and isomerisation^k^
cis-2-penten-1-ol	**C**_**5**_**H**_**10**_**O**	green-fruity^b^	13-alkoxy radicals^k^
cyclopentanol	**C**_**5**_**H**_**10**_**O**	unpleasant notes^d^	Hydroxyl pathway^k^
6-methylhepten-5-en-2-ol	**C**_**8**_**H**_**16**_**O**	Sweet, oily, green, coriander^k^	Plant metabolite^k^
1-hexanol	**C**_**6**_**H**_**14**_**O**	green-apple^b^; fruity, pungent, bitter^d^; grass, floral, aromatic^f^	13-hydroperoxides, LA metabolism, LOX ADH activity on C6 aldehydes^d^
trans-3-hexen-1-ol	**C**_**6**_**H**_**12**_**O**	fruity, soft^b^; astringent, bitter^e^	Isomerisation^d,k^
cis-3-hexen-1-ol	**C**_**6**_**H**_**12**_**O**	green^b^	13-hydroperoxides, LnA metabolism, LOX^d^
trans-2-hexen-1-ol	**C**_**6**_**H**_**12**_**O**	green-banana^b^	α-LnA metabolism, Isomerisation, LOX^d^
cis-2-hexen-1-ol	**C**_**6**_**H**_**12**_**O**	Green, leafy, fruity^k^	Isomerisation^d,k^
1-octene-3-ol	**C**_**8**_**H**_**16**_**O**	mushroom^a^; moistness-humidity^d^; mouldy^e^	Derivation from oct-1-en-3-one and short chain fatty acids^d^
3-methylheptan-1-ol	**C**_**8**_**H**_**18**_**O**	citrus^k^	Plant metabolite^k^
1-octanol	**C**_**8**_**H**_**18**_**O**	Waxy, green, citrus, aldehydic and floral with a sweet, fatty, coconut nuance^k^	Plant metabolite^k^
benzene methanol	**C**_**7**_**H**_**8**_**O**	Sweet, floral, fruity with chemical nuances^k^	Plant metabolite^k^
benzeneethanol	**C**_**8**_**H**_**10**_**O**	Sweet, floral, fresh and bready with a rosey honey nuance^k^	Plant metabolite; An antimicrobial, antiseptic and disinfectant^k^
1-hexadecanol	**C**_**16**_**H**_**34**_**O**	Waxy, clean, greasy, floral, oily^k^	reduction of palmitic acid^k^
butyraldehyde	**C**_**4**_**H**_**8**_**O**	Pungent, cocoa, green^k^	reductase using p-nitrobenzaldehyde^k^
2-methyl propanal	**C**_**4**_**H**_**8**_**O**	Fresh, aldehydic, floral, green, pungent^k^	*Saccharomyces cerevisiae* metabolite^k^
2-propenal	**C**_**3**_**H**_**4**_**O**	fruity, almond, cherry^k^	xenobiotic metabolite and a herbicide^k^
2-methyl butanal	**C**_**5**_**H**_**10**_**O**	unpleasant notes^d^; musty, rummy, nutty, cereal, caramellic, fruity^k^	plant metabolite, *Saccharomyces cerevisiae* metabolite^k^
3-methylbutanal	**C**_**5**_**H**_**10**_**O**	unpleasant notes^d^; fruity dry green chocolate nutty leafy cocoa^k^	plant metabolite, *Saccharomyces cerevisiae* metabolite^k^
pentanal	**C**_**5**_**H**_**10**_**O**	almond, malt^e^; winey, fermented^k^	Protein kinase catalytic activity^k^
hexanal	**C**_**6**_**H**_**12**_**O**	green, apple^b,e^; fruity, pungent, bitter^d^; grass^e^	HPL activity on 13-hydroperoxides, LA metabolism, LOX^d^
trans-2-pentenal	**C**_**5**_**H**_**8**_**O**	unpleasant notes^d^; green plant, grassy^h^	Autoxidation^d^; 13-alkoxy radicals^k^
cis-3-hexenal	**C**_**6**_**H**_**10**_**O**	fresh, green, herb^a,g^	HPL activity on 13-hydroperoxides, LnA metabolism, LOX^d^
heptanal	**C**_**7**_**H**_**14**_**O**	citrus, fatty, rancid^e;^ green, grassy, clover cilantro^k^	lipid oxidation^k^
trans-2-hexenal	**C**_**6**_**H**_**10**_**O**	fruity, pungent, bitter^d^; green, apple-like, almond, cut-grass^f^	α-LnA metabolism, Isomerisation, LOX^d^
octanal	**C**_**8**_**H**_**16**_**O**	lemon, fatty, green^e^; waxy, citrus, fruity^k^	plant metabolite^k^
trans-2-heptenal	**C**_**7**_**H**_**12**_**O**	unpleasant notes^d^; soap, fat, almond^e^	monounsaturated fatty acid catabolism with -oxo group^k^
nonanal	**C**_**9**_**H**_**18**_**O**	fat, citrus, green^e^; fatty, waxy, pungent^g^	reduction of the carboxy group of nonanoic acid^k^
trans, trans-2,4-hexadienal	**C**_**6**_**H**_**8**_**O**	ripe-fruit^b^; Sweet, green, waxy, aldehydic with fresh melon nuances^k^	polyunsaturated fatty acid catabolism^k^
trans, 2-octenal	**C**_**8**_**H**_**14**_**O**	unpleasant notes^d^; fatty, citrus, peel, spicy, cucumber^k^	antifungal agent
trans, trans-2,4-heptadienal	**C**_**7**_**H**_**10**_**O**	unpleasant notes^d^; fatty, green, oily, greasy^k^	Autoxidation^d^
decanal	**C**_**10**_**H**_**20**_**O**	fatty, soapy^h^; orange, peel, soap, tallow^j^	reduction of the carboxy group of capric acid (decanoic acid)^k^
trans, 2-nonenal	**C**_**9**_**H**_**16**_**O**	unpleasant notes^d^; tallow^j^; Green, cucumber, aldehydic, fatty with a citrus nuance^k^	monounsaturated fatty acid catabolism with -oxo group^k^
trans, 2-tridecenal	**C**_**13**_**H**_**24**_**O**	Aldehydic, citrus-Iike fatty, green and creamy^k^	plant metabolite^k^
cis/trans, 2-decenal	**C**_**10**_**H**_**18**_**O**	soapy, fatty^h^; tallow^j^; waxy, fatty, earthy, coriander, mushroom, green with a pork fat nuance^k^	Nature of some essential oils^k^
trans, trans, 2,4-nonadienal	**C**_**9**_**H**_**14**_**O**	unpleasant notes^d^ fatty^h^; melon, waxy^k^	Lipid oxidation^k^
2-undecenal	**C**_**11**_**H**_**20**_**O**	olive, fatty^h^; roasted, mango, waxy, citrus peel,^k^	Lipid oxidation^k^
trans, trans, 2,4-decadienal	**C**_**10**_**H**_**16**_**O**	unpleasant notes^d^; fatty, solvent^h^; fried, waxy, oily^j,k^	Lipid oxidation^k^
methyl benzene	**C**_**7**_**H**_**8**_	-	environmental pollution indicator & from insecticides^k^
ethyl benzene	**C**_**8**_**H**_**10**_	-	environmental pollution indicator & from insecticides^k^
xylene	**C**_**8**_**H**_**10**_	Plastic, geranium^k^	from insecticides and pharmaceuticals^k^
propyl benzene	**C**_**9**_**H**_**12**_	-	Simple form of phenylpropanoid lignin skeleton^k^
1,2,3-trimethyl benzene	**C**_**9**_**H**_**12**_	-	a neurotoxin and a plant metabolite; methylation of toluene and xylenes^k^
1,2,4-trimethyl benzene	**C**_**9**_**H**_**12**_	-	a neurotoxin and a plant metabolite; methylation of toluene and xylenes^k^
styrene	**C**_**8**_**H**_**8**_	Sweet, balsamic, floral, plastic^k^	a plant metabolite and a mouse metabolite, an acyclic olefin^k^
1,2,5-trimethyl benzene	**C**_**9**_**H**_**12**_	-	derivatization of benzene ring; oxidation of methyl groups^k^
vanillin	**C**_**8**_**H**_**8**_**O**_**3**_	Vanilla, sweet, spicy, phenolic^k^	a plant metabolite, an antioxidant and an anticonvulsant^k^
trans-α-bergamotene	**C**_**15**_**H**_**24**_	woody, warm^k^	a plant metabolite^k^
ethyl acetate	**C**_**4**_**H**_**8**_**O**_**2**_	winey, vinegary^c,d^; sticky, sweet^f^	Anaerobic fermentation^d^
butyl acetate	**C**_**6**_**H**_**12**_**O**_**2**_	sweet, ripe banana, tutti frutti, tropical and candy-like with green nuances^k^	Derivation of butan-1-ol^k^
ethyl propionate	**C**_**5**_**H**_**10**_**O**_**2**_	fruity^c^; etherial, fruity, sweet, winey, bubble gum, apple and grape nuances^k^	Derivation of ethanol^k^
hexyl acetate	**C**_**8**_**H**_**16**_**O**_**2**_	fruity^c^	13-hydroperoxides, LA metabolism, LOX^d^
methyl heptanoate	**C**_**8**_**H**_**16**_**O**_**2**_	pepper, sweet, fruit, green, orris, waxy, floral, berry^k^	methylation of heptadecanoic acid^k^
methyl octanoate	**C**_**9**_**H**_**18**_**O**_**2**_	waxy, green, sweet, orange, aldehydic, vegetable, herbal^k^	Formal condensation of the carboxy group of octanoic acid with the hydroxyl group of methanol^k^
octyl formate	**C**_**9**_**H**_**18**_**O**_**2**_	fruity, rose, orange, waxy, cucumber^k^	Derivation of octan-1-ol^k^
ethyl octadecanoate	**C**_**20**_**H**_**40**_**O**_**2**_	waxy^k^	Formal condensation between the carboxy group of octadecanoic (stearic) acid and the ydroxyl group of ethanol; a plant metabolite^k^
methyl nonanoate	**C**_**10**_**H**_**20**_**O**_**2**_	winey, waxy, green celery and pear, with an unripe fruit nuance^k^	formal condensation of methanol and nonanoic acid^k^
2-hydroxy methyl benzoate	**C**_**8**_**H**_**8**_**O**_**3**_	Sweet, creamy, vanilla-like, spicy, woody^i^	Nature of fruit^i^
cis-3-hexene-1-ol acetate	**C**_**8**_**H**_**14**_**O**_**2**_	banana-like, green, fruity, floral, ester^f^	13-hydroperoxides, LnA metabolism, LOX, AAT activity on C6 alcohols^d^
Hexanoic acid, 1-methylethyl ester	**C**_**9**_**H**_**18**_**O**_**2**_	fruity, pineapple, berry^k^	a plant metabolite^k^
2-pentyl furan	**C**_**9**_**H**_**14**_**O**	fruity, green, earthy, beany, vegetable^k^	*Aspergillus fumigatus* infections; catabolic reactions^k^
2-ethyl furan	**C**_**6**_**H**_**8**_**O**	sweet, burnt, earthy, malty^k^	a constituent of numerous plant species^k^
octane	**C**_**8**_**H**_**18**_	gasoline^i^	Xenobiotic^i^
1,1-dimethyl-2-(2-methyl-2-propenyl)-cyclopropane	**C**_**9**_**H**_**16**_	-	fragmentation^k^
decane	**C**_**10**_**H**_**22**_	-	fragmentation^k^
2-ethyl, 6-methyl, 1,5-heptadiene	**C**_**10**_**H**_**18**_	-	fragmentation^k^
3-ethyl, 1,5-octadiene	**C**_**10**_**H**_**18**_	-	a plant metabolite^k^
4,8-dimethyl, 1,7-nonadiene	**C**_**11**_**H**_**20**_	-	a constituent of aromatic oils^k^
4,5-dimethyl, 2,6-octadiene	**C**_**10**_**H**_**18**_	-	fragmentation^k^
2-methyl, 6-methylene, 2-octene	**C**_**10**_**H**_**18**_	-	fragmentation^k^
trans-3-octadecene	**C**_**18**_**H**_**36**_	-	fragmentation^k^
3-carene	**C**_**10**_**H**_**16**_	Citrus, sweet, terpenic, fir needle^k^	Dimerization of C5 molecules^d^; a plant metabolite^k^
docosane	**C**_**22**_**H**_**46**_	waxy^k^	a plant metabolite ^**k**^
tetracosane	**C**_**24**_**H**_**50**_	-	a plant metabolite ^**k**^
squalene	**C3**_**0**_**H**_**50**_	floral^k^	olive metabolite^k^
pentacosane	**C**_**25**_**H**_**52**_	waxy^k^	a plant metabolite ^**k**^
galangin	**C**_**15**_**H**_**10**_**O**_**5**_	bitter^k^	7-hydroxyflavonol with additional hydroxy groups; an antimicrobial agent; a plant metabolite^k^
2-propanone	**C**_**3**_**H**_**6**_**O**	solvent, ethereal, apple, pear^k^	occurs naturally in plants, trees, forest fires, vehicle exhaust^k^
2-butanone	**C**_**4**_**H**_**8**_**O**	ethereal, fruity, camphoraceous^k^	a bacterial metabolite^k^
2-pentanone	**C**_**5**_**H**_**10**_**O**	Sweet, fruity, ethereal, wine, banana, woody^k^	a plant metabolite ^**k**^
3-pentanone	**C**_**5**_**H**_**10**_**O**	sweet^b^; ethereal, acetone^k^	*Triatoma brasiliensis, Triatoma infestans* infections
4-methyl, 2-pentanone	**C**_**6**_**H**_**12**_**O**	Sharp, solvent-like with green, herbal, fruity and dairy nuances^k^	**-**
1-penten-3-one	**C**_**5**_**H**_**8**_**O**	sweet, strawberry, pungency, metallic^b,c,d^; green, strawberry, sharp^f^	Prolonged contact with metal surface^d^; 13-alcoxy radicals^k^
2-heptanone	**C**_**7**_**H**_**14**_**O**	wet soil^c^	Fungal activity^c^
2-octanone	**C**_**8**_**H**_**16**_**O**	unpleasant notes^d^; musty, ketonic, cheesy, earthy, dairy	Lipid oxidation^d^
2-pentadecanone	**C**_**15**_**H**_**30**_**O**	Fatty, spicy, floral^k^	a plant metabolite ^**k**^
5-ethyl-(5h)-furan-2-one	**C**_**6**_**H**_**8**_**O**_**2**_	Spicy^k^	a plant metabolite ^**k**^
6-methyl, 5-heptene-2-one	**C**_**8**_**H**_**14**_**O**	green-fruity, grass^e^	a plant metabolite ^**k**^
5-methyl, 4-hexene-3-one	**C**_**7**_**H**_**12**_**O**	-	a plant metabolite^k^
acetic acid	**C**_**2**_**H**_**4**_**O**_**2**_	winey-vinegary^c,d^	Anaerobic fermentation^d^
propanoic acid	**C**_**3**_**H**_**6**_**O**_**2**_	unpleasant notes^d^; rancid, soya, pungent^e^	Anaerobic fermentation^d^
butanoic acid	**C**_**4**_**H**_**8**_**O**_**2**_	unpleasant notes^d^ rancid, soya, pungent^e^	Anaerobic fermentation^d^
hexanoic acid	**C**_**6**_**H**_**12**_**O**_**2**_	oily^h^	Caproic acid is found naturally in various plant^k^
heptanoic acid	**C**_**7**_**H**_**14**_**O**_**2**_	Cheesy, waxy, sweaty, fermented, pineapple, fruity^k^	a C7, straight-chain fatty acid that contributes to the odour of some rancid oils^k^
octanoic acid	**C**_**8**_**H**_**16**_**O**_**2**_	Fatty, waxy, rancid, oily, cheesy^k^	an antibacterial agent; a conjugate acid of an octanoate^k^
nonanoic acid	**C**_**9**_**H**_**18**_**O**_**2**_	Waxy, dirty and cheesy with a cultured dairy nuance^k^	herbicide; antifungal^k^
octadecanoic acid	**C**_**18**_**H**_**36**_**O**_**2**_	Odourless, mild, fatty, waxy^k^	Nature of some essential oils^k^
6-octadecenoic acid	**C**_**18**_**H**_**34**_**O**_**2**_	-	Nature of some essential oils^k^
cis-9-octadecenoic acid	**C**_**18**_**H**_**34**_**O**_**2**_	Fatty, vegetable oil^k^	Nature of some essential oils^k^
trans-9-octadecenoic acid	**C**_**18**_**H**_**34**_**O**_**2**_	-	Nature of some essential oils^k^
α-copaene	**C**_**15**_**H**_**24**_	sweet, fruity^h^; woody, spicy, honey^k^	a plant metabolite^k^
zingiberene	**C**_**15**_**H**_**24**_	floral^h^; spicy, fresh, sharp^k^	a plant metabolite^k^
δ-curcumene	**C**_**15**_**H**_**24**_	herbal k	a plant metabolite^k^
α-Sesquiphellandrene	**C**_**15**_**H**_**24**_	floral^h^	a plant metabolite^k^
trans- caryophyllene	**C**_**15**_**H**_**24**_	wood, oak, dry^a^	a plant metabolite^k^
funebrene	**C**_**15**_**H**_**24**_	cedarwood, woody^k^	a plant metabolite^k^
β- himachalene	**C**_**15**_**H**_**24**_	-	a plant metabolite^k^
valencene	**C**_**15**_**H**_**24**_	mint, orange blossom^h^	a plant metabolite^k^
α-muurolene	**C**_**15**_**H**_**24**_	Herbal, woody, spicy^k^	a plant metabolite^k^
α-farnesene	**C**_**15**_**H**_**24**_	floral, green plant, herb^h^	Natural coating material of fruits for defencing against to fungal attack^i^
cadidene	**C**_**15**_**H**_**24**_	wood, fragrant, flowery^a^	a plant metabolite^k^
bisabolene	**C**_**15**_**H**_**24**_	balsamic, woody^k^	a plant metabolite^k^
α-pinene	**C**_**10**_**H**_**16**_	pine, fragrant, fresh^a^; woody, herbal, spicy, tropical^k^	Dimerization of C5 molecules^d^; a plant metabolite^k^
β-myrcene	**C**_**10**_**H**_**16**_	balsamic^a^	Dimerization of C5 molecules^d^; a plant metabolite^k^
limonene	**C**_**10**_**H**_**16**_	sweet, citrus^a^	Dimerization of C5 molecules^d^; a plant metabolite^k^
trans-ocimene	**C**_**10**_**H**_**16**_	herb^a^	Dimerization of C5 molecules^d^; a plant metabolite^k^
α-terpinene	**C**_**10**_**H**_**16**_	lemon, wood^a^	Dimerization of C5 molecules^d^; a plant metabolite^k^
linalool	**C**_**10**_**H**_**18**_**O**	lilac, lavender^f^	Phenolic-protein interactions^f^

LOX: lipoxygenase enzymatic pathway; LA: Linoleic acid; LnA: Linolenic acid. HPL: hydroperoxide lyase; ADH: Alcohol dehydrogenase; AAT: alcohol acetyl transferase. ^**a**^Song and Liu (2018); ^**b**^Ridolfi et al. (2002); ^**c**^FEMA (2020); ^**d**^Boskou (2012); ^**e**^Aparicio-Ruiz et al. (2018); ^**f**^Genovese et al. (2018); ^**g**^Kalua et al. (2007); ^**h**^Kesen et al. (2013); ^**i**^Abbatangelo et al. (2019); ^**j**^Zhu et al. (2016); ^**k**^ web sites (flavornet.org; pubchem.ncbi.nlm.nih.gov; TGSC Information System).

**Table 5:** Volatile profiles of Ayvalık VOOs, %.

Codes	Aldehydes		Ketones		Esters		Alcohols		Organic Acid	Terpene	Benzene Ringed	Furan Ringed	Hydrocarbons
	Total	*by LOX*	Total	*by LOX*	Total	*by LOX*	Total	*by LOX*					
GO1	28.40 h	78.14 H	4.03 d	46.17 H	0.71 j	46.11 A	15.01 c	62.64 F	3.21 e	8.53 h	2.36 m	0.73 h	37.02 e
GO2	30.03 g	84.82 C	3.57 e	43.59 J	1.28 g	2.91 L	13.76 e	62.61 F	3.29 d	11.15 g	2.79 i	1.35 c	32.78 g
GO3	31.19 e	76.32 I	3.10 f	34.09 L	0.82 i	42.46 C	13.72 f	60.92 G	6.61 b	5.70 k	10.40 a	1.25 d	27.22 h
GO4	23.94 j	81.32 G	9.48 a	33.04 M	0.31 k	23.08 D	26.71 a	57.78 H	2.81 f	6.89 i	10.30 b	0.54 j	19.03 i
GO5	23.28 k	78.12 H	2.51 j	42.18 K	0.85 i	4.54 I	15.20 b	48.63 K	2.55 g	14.58 d	7.76 c	0.53 j	32.74 g
GO6	31.19 e	61.84 L	3.11 f	52.28 F	0.69 j	6.55 H	12.86 g	62.94 E	2.79 f	4.15 l	2.46 l	1.14 e	41.61 d
GO7	35.00 d	72.89 J	2.31 k	73.95 C	2.50 c	4.32 J	13.99 d	52.24 I	8.54 a	12.99 e	2.67 j	3.75 a	18.23 j
GO8	7.50 m	83.41 E	0.43 m	0.00 N	0.02 l	0.00 N	8.05 k	65.92 D	0.70 i	0.32 n	2.56 k	0.09 k	80.32 a
GO9	27.62 i	83.51 D	1.87 l	74.32 B	0.92 h	3.01 K	12.20 h	38.73 L	2.00 h	2.51 m	4.53 f	1.06 f	47.30 b
GO10	35.77 c	65.87 K	3.02 g	44.21 I	2.04 d	11.86 F	5.80 l	85.42 B	4.52 c	32.48 b	4.45 g	0.01 l	11.90 l
GO11	21.20 l	87.36 A	2.61 i	68.85 D	1.37 f	1.47 M	2.24 n	50.28 J	0.28 j	38.14 a	1.10 n	0.00 l	33.06 f
GO12	30.90 f	82.44 F	2.69 h	55.87 E	1.78 e	44.98 B	10.13 i	34.53 M	2.08 h	6.14 j	3.58 h	0.67 i	42.04 c
GO13	47.45 a	86.66 B	5.31 b	46.97 G	6.04 b	10.84 G	8.22 j	76.11 C	0.00 k	12.45 f	5.24 e	0.87 g	14.41 k
GO14	42.65 b	83.43 E	4.85 c	91.11 A	6.49 a	12.66 E	5.18 m	92.06 A	0.74 i	21.53 c	5.67 d	1.48 b	11.42 m

LOX: lipoxygenase enzymatic pathway, Standard Deviations ≤0.2.

Means within a column with different letters are significantly different (P ≤ 0.05).

Aldehydes were found the most abundant volatiles in "Ayvalık" VOOs. The aldehyde compounds (n:24) were detected with a total concentration of 11.77 ppm. The terpenes (n:18) were detected in a total amount of 8.08 ppm. So, terpenes were determined as the second abundant volatile group of "Ayvalık" VOOs. The alcohols (n:21) were isolated; however not all of them was generated by LOX reactions. Their concentration was 7.07 ppm. The benzene ringed compounds (n:10) were identified with a total amount of 2.83 ppm. 11 ketone, 11 acid, 12 ester, and 3 furan compounds were defined by approximately 1.63, 1.74, 0.71 and 0.23 ppm, respectively. Guclu et al. (2016) reported for Ayvalık VOOs obtained from different growing area (Mersin province) in Turkey that 6.3 ppm aldehyde, 6.2 ppm alcohol, 2.3 ppm terpene, 0.2 ppm ester, 0.002 ppm butanoic acid presence.

**Figure 3:**
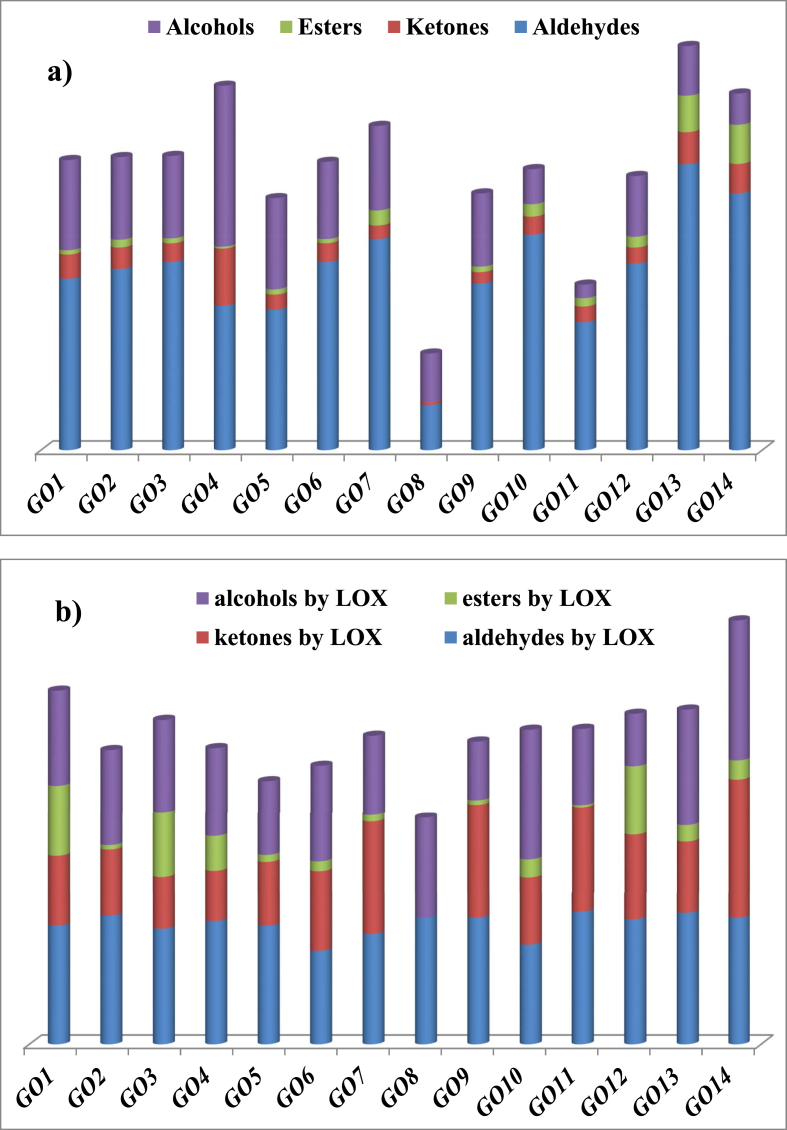
Volatile profiles of Ayvalık VOOs; a) total amount (ppm) of volatiles b) total percentage of volatiles generated via LOX pathway.

With another perspective, isolated organic volatile compounds namely, aldehydes, ketones, esters, alcohols, acids, terpenes, benzene and furan ringed contents, and straight hydrocarbons were totally found in this study that 100.38 ppm for GO1, 57.43 ppm for GO2, 81.62 ppm for GO3, 18.6 ppm for GO4, 135.09 ppm for GO5, 36.36 ppm for GO6, 41.39 ppm for GO7, 55.43 ppm for GO8, 50.67 ppm for GO9, 86.67 ppm for GO10, 42.92 ppm for GO11, 20.57 ppm for GO12, 13.74 ppm for GO13 and 12.17 ppm for GO14 growing areas, respectively. For "Ayvalık" VOOs, it could be seen from Figure 3a that aldehydes and alcohols were the most detected volatile group generated by LOX. At the same time, ester and ketone concentration were obviously varied between geographical origins. C5 and C6 aldehydes, ketones, alcohols and esters produced by LOX pathway gains to VOO fresh-green notes (cut grass, unripe fruit, almond), green-fruity notes (green, sweet, strawberry, herbal), green-fruity notes (leaf, grass, bitter, banana), fruity-floral notes (sweet, banana-tomato, flowery, species), respectively (Table 4).

As also shown at Figure 3b, GO1 and GO3 had well-balanced odour based on LOX volatiles. There were no ketone and ester compounds in volatile fraction of GO8 formed by LOX even any harvest year. Moreover, a little ester content was isolated from GO2, GO9 and GO11. The highest content of aldehydes was detected at GO11, the highest content of ketones at GO14, the highest content of esters at GO1, the highest content of alcohols at GO14 geographical origin. Most of terpene compounds stem from olive fruit as a plant metabolite (Table 4). In this study, the highest terpene contents were defined at GO11 and GO10 respectively (Table 5). These volatile compounds attributed with woody, herbal, spicy, sharp and floral odours (Table 4). It was characteristically found that "Ayvalık" VOOs contains 7.50–47.45% aldehydes, 0.32–38.14% terpenes, 2.24–26.71% alcohols, 1.10–10.40% benzene ringed compounds, 0.43–9.48% ketones, 0.00–8.54% organic acids, 0.02–6.49% esters, 0.00–3.75% furans. The rest of those had contained straight chain-hydrocarbons and some other fragmentation products. It was also observed that 61.84–87.36% of aldehydes, 0.00–91.11% of ketones, 0.00–46.11% of esters and 34.53–92.06% of alcohols were generated only by LOX pathway among investigated harvest seasons (Table 5).

## 4 Conclusions

A notable and comprehensive data set was generated including quality indices, oxidative properties, fatty acid and volatile profiles of “Ayvalık” VOOs by this presented research. 125 different volatile compounds were isolated and amounts were calculated. Their generation mechanisms were chemically defined. Aldehydes, terpenes and alcohols were found the most three abundant volatile groups. Overall high-quality “Ayvalık” VOOs can be described as full of positive attributes such as fresh, green, fruity, flowery notes. The major fatty acids were oleic, palmitic, linoleic, stearic and palmitoleic acids, respectively. Quality parameters and profile of fatty acids and volatiles isolated from “Ayvalık” EVOO showed a strong link with its geographical roots. It was expected that by this way, a rapid acceleration in the number of new geographical registrations, which is one of the most competitive economic strategies for value-added VOOs, linked “Ayvalık” variety.

## Declarations

### Author contribution statement

Didar Üçüncüoğlu: Conceived and designed the experiments; Performed the experiments; Analyzed and interpreted the data; Contributed reagents, materials, analysis tools or data; Wrote the paper.

Dilek Sivri-Özay: Conceived and designed the experiments; Contributed reagents, materials, analysis tools or data; Wrote the paper.

### Funding statement

This work was financially supported by the Republic of Turkey, 10.13039/501100006697Ministry of Agriculture and Forestry, General Directorate of Agricultural Research and Policies (TAGEM,) Research & Development Project (R&D 12–26), Turkey. The volatile analysis were funded by 10.13039/501100005378Hacettepe University Scientific Research Projects Coordination Unit (Project, 1933), Turkey.

### Competing interest statement

The authors declare no conflict of interest.

### Additional information

No additional information is available for this paper.
